# Distributed Raman Amplification for Fiber Nonlinearity Compensation in a Mid-Link Optical Phase Conjugation System

**DOI:** 10.3390/s22030758

**Published:** 2022-01-19

**Authors:** Mingming Tan, Paweł Rosa, Tu T. Nguyen, Mohammad A. Z. Al-Khateeb, Md. Asif Iqbal, Tianhua Xu, Feng Wen, Juan D. Ania-Castañón, Andrew D. Ellis

**Affiliations:** 1 Aston Institute of Photonics Technologies, Aston University, Birmingham B4 7ET, UK; nguyentu.hcmuns@gmail.com (T.T.N.); elkhateeb.eng@gmail.com (M.A.Z.A.-K.); mdasif.iqbal@bt.com (M.A.I.); andrew.ellis@aston.ac.uk (A.D.E.); 2 National Institute of Telecommunications, Szachowa 1, 04-894 Warsaw, Poland; 3 School of Engineering, University of Warwick, Coventry CV4 7AL, UK; tianhua.xu@warwick.ac.uk; 4 School of Information and Communication Engineering, University of Electronic Science and Technology of China, Chengdu 611731, China; fengwen@uestc.edu.cn; 5 Instituto de Óptica “Daza de Valdés”, 28006 Madrid, Spain; jd.ania@csic.es

**Keywords:** Raman amplification, coherent fiber optic communications, optical phase conjugation

## Abstract

In this paper, we review different designs of distributed Raman amplifiers which have been proposed to minimize the signal power profile asymmetry in mid-link optical phase conjugation systems. We demonstrate how the symmetrical signal power profiles along the fiber can be achieved using various distributed Raman amplification techniques in the single-span and more realistic multi-span circumstances. In addition, we show the theoretically predicted results of the Kerr nonlinear product reduction with different Raman techniques in mid-link optical phase conjugator systems, and then in-line/long-haul transmission performance using numerical simulations.

## 1. Introduction

Mid-link optical phase conjugation (OPC) has been used to compensate both linear (e.g., chromatic dispersion) and the nonlinear (e.g., the Kerr nonlinearity) impairments of the optical fiber, which can significantly enhance the maximum transmission distance or the data capacity, particularly for a relatively long-haul transmission system [1,2,3,4,5,6,7,8,9,10,11,12,13,14,15,16,17,18,19,20,21,22,23,24,25,26,27,28]. There are a few limiting factors in the optical fiber link which constrain the efficiency of combating the nonlinear impairment in a mid-link OPC system, such as the chromatic dispersion slope and the signal power profile along the fiber [1,2,3]. Erbium-doped fiber amplifiers (EDFAs) are the most widely used amplification technique to compensate the loss in the optical fiber, but typically for a mid-link OPC system using the EDFAs in the link demonstrates that the either the maximum reach is not significantly extended, or the performance gain is modest [4,5,6,7,8,9], even when dispersion management is employed [10,11]. This is because of the lack of symmetrical signal power profiles before and after a mid-link OPC [12,13]. With increased availability of high power semiconductor pump lasers, and increased confidence in fiber power handling, Raman amplifiers are extensively used in unrepeatered submarine systems and terrestrial transmission systems [29,30], significantly improving the signal-to-noise ratio. Distributed Raman amplification (DRA) essentially uses the transmission fiber as the Raman gain medium and provides the signal amplification along the fiber, in comparison with an EDFA which is a lumped amplifier using a short Erbium-doped fiber as the gain medium. The design of DRA is highly flexible: Pump wavelength can be chosen and adjusted with fiber Bragg gratings (FBGs). A purposefully built DRA can also improve the power symmetry of the optical fiber link, enabling enhanced efficiency of nonlinearity compensation, and therefore the overall transmission performances [31,32,33]. Thus, the DRA not only provides an improved signal-to-noise ratio (SNR) without OPC, but also gives a large margin in the transmission performance (maximum reach, BER or data capacity) improvement when using an OPC to compensate a significant portion of nonlinear product in the transmission systems. In [12,13], K. Solis-Trapala et al. investigated the signal power symmetry and transmission performance of the bidirectional pumping over dispersion flattened non-zero dispersion-shifted fiber (NZDSF) in a mid-link OPC system, but the selected Raman scheme was restricted to first-order bidirectional pumping with similar pump power from both directions. The conclusion was that the shortest fiber length (25 km) gave the highest signal power profile symmetry. This optimized fiber length of 25 km is too short for realistic fiber spans in current optical transmission systems.

In this paper, we review various designs of distributed Raman amplification schemes aimed at improving the symmetry of the link for the transmission systems with mid-link optical phase conjugators. We show the optimized Raman amplification designs over single fiber span and multiple fiber spans (two) which can both demonstrate symmetry levels of above 93%. For the single-span link with a 50 km standard single mode fiber (SSMF) with backward Raman pumping only, the dual-order DRA can achieve 97% signal power profile symmetry and 39 dB nonlinear product compensation. This leads to more nonlinear product compensation, 12 dB higher, in comparison with conventional first-order DRA. For longer span links (e.g., 62 km), bidirectional Raman pumping is required to achieve good symmetry. A distributed Raman scheme based on the random distributed feedback laser architecture has been shown to maximize the signal power profile symmetry (97% symmetry) without introducing significant transmission performance penalty from relative intensity noise (RIN) of forward pumping. This proposed scheme can give 37.6 dB nonlinear product compensation, comparable to the 39 dB over 50 km dual-order backward pumping Raman scheme. Furthermore, for multi-fiber-span (i.e., 2 × 50 km SSMF) links, the impact of loss between the spans is crucial when optimizing the symmetry. The conventional dual-order backward-pumped DRA can give only ~66% signal power profile symmetry, achieving ~17 dB nonlinear product compensation. An over-pumped first span using the net gain to compensate the loss between spans would help improve the symmetry to 81% leading to a 25 dB nonlinear product reduction. The best solution is that a 25 cm erbium-doped fiber (EDF) is embedded in the conventional dual-order DRA, but the Raman pumps are used to pump the EDF and so compensate the loss between the two spans, which enables the overall link symmetry to be optimized to 93.9% providing up to 32 dB nonlinear product compensation with 50 km per span. In addition, we demonstrate that in a relatively short transmission system (100–200 km), the higher signal power profile symmetry, the higher the nonlinear threshold (up to 9 dB) when using OPC. We also show, in the numerical simulations, a Q^2^ factor improvement of approximately 8 dB in the long-haul transmission systems (2000 km) using the optimized EDF-assisted Raman amplified spans in the link.

## 2. Optimized Distributed Raman Amplification Design over Single Fiber Span

As the stimulated Raman scattering allows a Stokes shift (13 THz) from the pump to the signal which gives more flexibility for the choice of the pump wavelengths and the Raman gain fiber, Raman amplification is highly configurable. For example, in Reference [30], a cascaded third-order Raman pump was used in an unrepeatered transmission experiment. In Reference [34], the authors presented a sixth-order Raman pump configuration. However, the commonly used Raman schemes are based on first-order and second-order Raman amplifiers which have relatively configurations, higher pump-signal conversion efficiency and consequently lower cost [35,36,37,38,39,40,41,42,43]. Using only forward Raman pumping is generally not feasible for transmission systems because firstly, it introduces significant RIN-related penalty to the transmitted signal from the pump, and secondly the signal suffers high fiber nonlinearity due to high signal power near the input sections of the transmission span [35,36,37,38,39,40,41,42,43]. Therefore, in this paper, we mainly focus on the designs of first-order and second-order distributed Raman amplification schemes using backward or bidirectional pumping which requires the RIN penalty mitigation technique.

### 2.1. Distributed Raman Amplification with Backward Pumping Only

**Figure 1:**
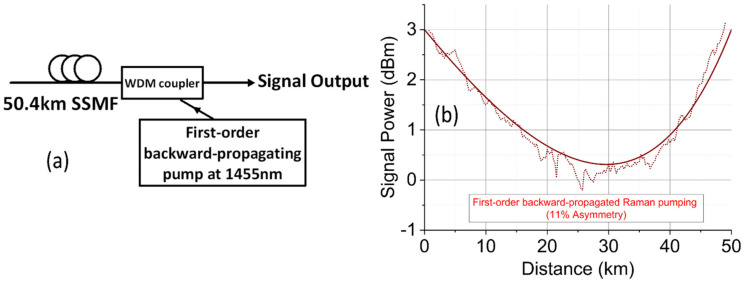
(**a**) First-order backward pumping; (**b**) signal power profiles along the fiber.

Figure 1a shows the schematic diagram of the conventional first-order backward-pumped DRA, including the 50.4 km SSMF pumped by a fully depolarized fiber laser at 1455 nm. Because the fiber length is only ~50.4 km mainly for short reach or multiple spans in long-haul transmission systems, the span loss was fully compensated which means that the pump power was commonly set to achieve zero dB net gain [43,44,45,46,47,48]. Figure 1b shows both experimentally measured and numerically simulated signal power profiles along the fiber using the modified optical time domain reflectometer (OTDR) technique [44]. The symmetry/asymmetry level of the signal power profile is based on the calculation method in [12,13], which gave 89% symmetry (11% asymmetry) for this Raman scheme.

**Figure 2:**
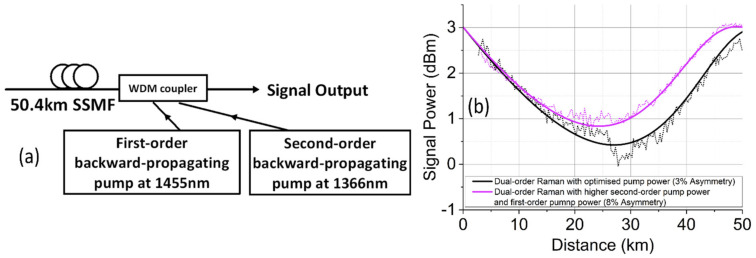
(**a**) Dual-order backward pumping; (**b**) signal power profiles along the fiber.

Figure 2a shows a dual-order backward-pumping DRA scheme including both a 1365 nm Raman fiber laser and a 1455 nm pump seed [49,50]. Figure 2b shows the simulated and experimentally measured signal power profiles along the fiber. The use of the dual-order BW-pumping scheme can improve the symmetry of signal power profile, but it requires the optimization of the first- and second-order pump power [23]. As shown in Figure 2b, to achieve 97% symmetry, the first- and second-order pump power was set to ~100 and ~330 mW respectively. When the second-order pump power is increased beyond this optimum, the signal power symmetry degrades to 92% with ~33 and ~600 mW first- and second-order pump powers, respectively. This asymmetry accumulation was mainly because the signal power was increased near the signal output, as the signal gain was pushed into the middle of the fiber span using higher second-order pump power [23], whilst for lower second-order pump powers, the asymmetry approaches that of the first-order pumped configuration.

**Figure 3:**
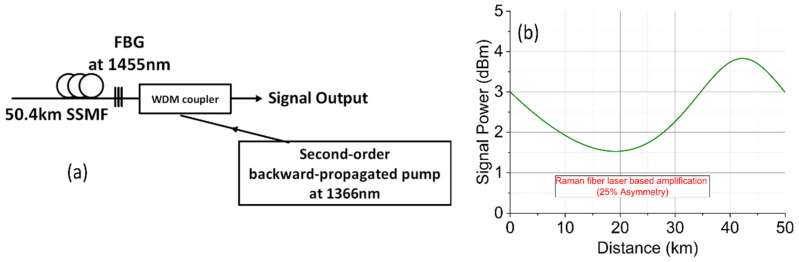
(**a**) Raman-fiber-laser-based amplification with second-order pumping; (**b**) simulated signal power profiles.

As the alternative to the dual-order Raman scheme, the pump seed at 1455 nm can be replaced by a highly reflective fiber Bragg grating (FBG) as shown in Figure 3a [35]. In this case, a random distributed feedback (DFB) fiber laser at 1455 nm was generated due to the distributed Rayleigh backscattering (originating from the SSMF) and the fixed reflection of the FBG [35,36,37]. This scheme is more cost-effective in comparison with the dual-order scheme, as no active pump at 1455 nm is required. However, in terms of flexibility when optimizing the signal power symmetry, a random DFB fiber laser is not flexible compared to independent separate pumps as a minimum second-order pump power is determined by the grating reflectivity and the stimulated Brillouin scattering (SBS) coefficient. The same parameters also determine the ratio of first to second-order pump power. For a typical grating reflectivity of 99%, the power symmetry was simulated to be only 75% for the 50.4 km SSMF span.

**Figure 4:**
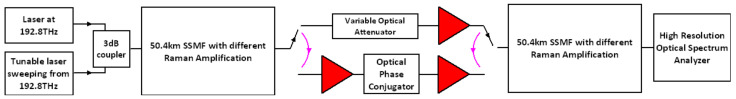
Schematic diagram of nonlinear product measurement using dual-order DRA in mid-link OPC systems.

**Figure 5:**
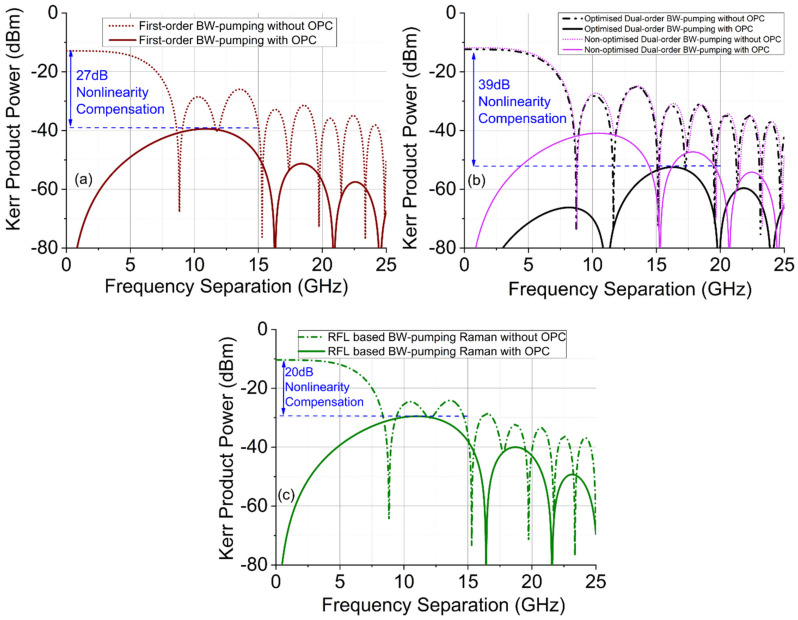
Theoretical predication of nonlinear Kerr product power as a function of frequency separation between the two CW lasers over 50 km SSMF. (**a**) First-order Raman amplification. (**b**) Dual-order Raman amplification with optimized pump power and non-optimized pump power). (**c**) Random Raman-fiber-laser-based amplification.

We have theoretically predicted the power of the nonlinear Kerr product generated by two co-polarized CW lasers (3 dBm each) along a 50.4 km backward-pumped DRA system with and without mid-link OPC. Figure 4 shows the configuration used to predict the power of the Kerr nonlinear product as a function of frequency separation of the two lasers [23]. Figure 5 demonstrates the nonlinear product power versus laser frequency separation using the three different Raman schemes described above without and with mid-link OPC. For the conventional first-order DRA, the nonlinear product power without OPC was up to −11.5 dBm at low frequency ranges. With OPC, the peak Kerr product power was suppressed by 27 dB. Using an optimized dual-order Raman scheme would improve the symmetry of signal power distributions and therefore further decrease the nonlinear Kerr product using the mid-link OPC. In Figure 5b, using the non-optimized pump power configuration (600 mW second-order pump power), the peak Kerr product power reduction was improved to ~30 dB, but once the pump power was optimized to maximize the symmetry (330 mW second-order pump power), the Kerr product reduction was increased to 39 dB. The random-fiber-laser-based scheme gave the lowest symmetry, and therefore the reduction in Kerr product power with OPC was limited to ~20 dB, as illustrated in Figure 5c.

An inline coherent transmission experiment was conducted by replacing the two CW lasers shown in Figure 4 with a 256 Gb/s DP-16QAM (32 GBaud) signal (centered at 194.8 THz). After the transmission, the signal or its conjugate (centered at 194.65 THz) was amplified with an EDFA as the receiver amplifier before being detected by a polarization-diverse coherent receiver (100 GSa/s, analog bandwidth of 33 GHz). Commercial digital signal processing (DSP) software was used to process the captured data from the scope with Q^2^ factors calculated from the bit-error-rate of 500,000 bits.

**Figure 6:**
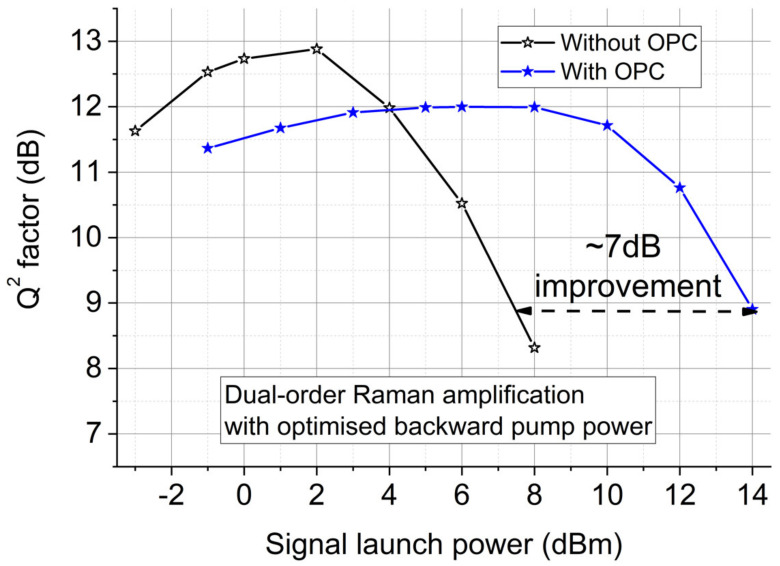
Experimentally measured Q^2^ factors versus signal launch power in inline transmission systems without/with OPC using the optimized dual-order DRA scheme over 2 × 50 km SSMF.

As shown in Figure 6, ~5 dB improvement in the nonlinear threshold and ~7 dB increases in launch power for a fixed Q^2^ in the nonlinear region are achieved when using the nearly perfect (~97%) signal power symmetry provided by dual-order BW-pumping DRA. The optimum Q^2^ factor was reduced because of the short transmission distance (100 km) and the additional ASE noise added by the inclusion of the OPC. However, using the optimized OPC in a long-haul transmission system has been proven to significantly improve the system performance, since the transceiver and OPC noise are negligible compared with the accumulated link noise which is the majority of the linear noise [23].

### 2.2. Distributed Raman Amplification with Bidirectional Pumping

In our optimization of signal power profiles for mid-link OPC we consider three different distributed Raman amplification schemes, all of them bidirectionally pumped. Simulations are performed for each configuration, obtaining signal power excursion for different pump power ratios and span lengths using the tried and tested model fully described in [38] with the boundary conditions corresponding to each of the cases under consideration and assuming fully depolarized pumps as well as room temperature operation. Noise calculations are based on a 0.1 nm bandwidth. The coefficients for Raman gain and attenuation at the different wavelengths involved were extracted from measurements for SSMF [38], whereas the Rayleigh backscattering coefficients at 1366 and 1455 nm and the frequency of the signal are assumed to be 1.0 × 10^−4^, 6.5 × 10^−5^ and 4.5 × 10^−5^ km^−1^, respectively.

**Figure 7:**
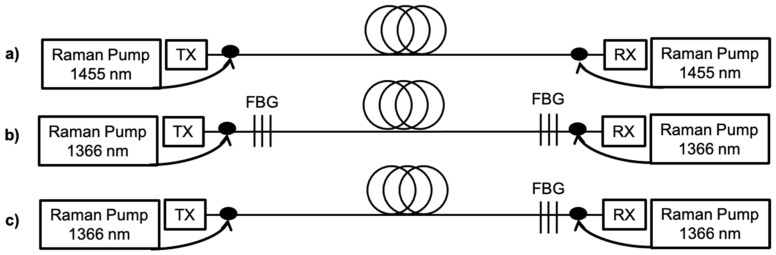
Schematic design of (**a**) first-order Raman, (**b**) second-order ultra-long fiber laser and (**c**) second-order random DFB Raman laser amplifiers.

The first case considered (Figure 7a) corresponds to a conventional bidirectionally pumped first-order Raman amplifier, with pumps at 1455 nm that amplify the signal through the first Stokes shift.

The second case corresponds to an ultra-long Raman fiber laser (URFL) amplifier (Figure 7b), that provides second-order pumping from single-wavelength pumps. In such an amplifier, the initial Raman fiber laser pumps operate at 1366 nm, that is, downshifted by two Stokes shift with respect to the frequency of the signal. Highly reflective (99%) fiber Bragg gratings (FBGs) centered at 1455 nm with a bandwidth of 200 GHz are located at both ends of the transmission line to back-reflect the first Stokes-shifted radiation at 1455 nm into the long cavity. Once a threshold of ~0.8 W pump power is reached, the cavity forms a stable ultra-long laser that at that amplifies the signal around 1550 nm. This approach presents the advantage of having modifiable gain bandwidth and profiles by selecting appropriate FBGs, instead of requiring an active seed at the intermediate Stokes. In this case the reflectivity of the FBGs was chosen to be high to maximize pump-to-signal power conversion efficiency [38,39,40,41].

The third and final approach uses a random distributed feedback Raman laser amplifier (Figure 7c). This is another fiber laser amplifier similar in part to the scheme shown in Figure 3. This is significantly different from a closed cavity with two FBGs. The scheme is essentially bidirectional Raman pumping using a half-open-cavity design with a single high-reflectivity FBG at 1455 nm located at the end of the span that reflects the 1455 nm Stokes in the backward direction. The second-order pump in front of the span, does not create a seed traveling in forward direction (either by inserting an FBG or an active seed) but rather amplifies the seed created at the end of the span. The lack of an FBG on the side of the forward pump reduces the RIN transfer to the 1455 nm Stokes in exchange for a reduction in conversion efficiency in comparison to the other two proposed setups [35]. This reduced interaction between the signal and the forward pumping is particularly important, as the RIN transfer from high-power forward pumps can be a limiting factor in data transmission [42,43].

**Figure 8:**
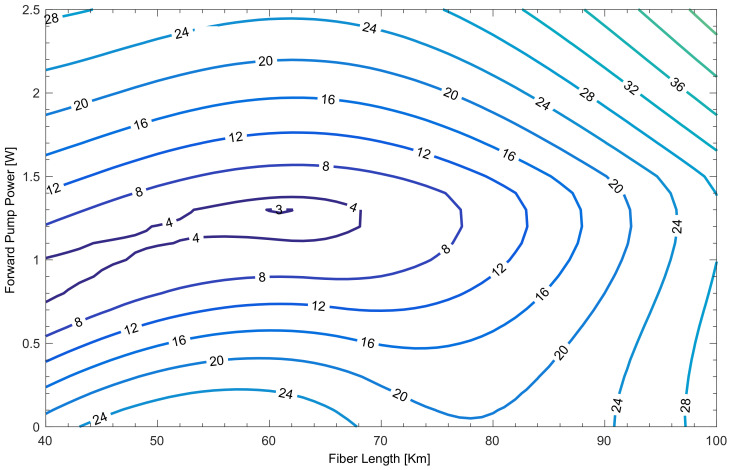
Signal power asymmetry (%) as a function of different span lengths and pump powers (backward pump power was adjusted to give 0 dB net gain).

In order to perform a comparison of signal power asymmetry between the three proposed configurations, we simulated power profiles of a single channel at 1545 nm with a fixed launch power of 0 dBm. For each value of the forward pump power (FPP) and span lengths ranging from 40 to 100 km, the backward pump was adjusted to provide 0 dB net gain. Signal power asymmetry was calculated as in [12,13]. Full results using random DFB laser amplification (Figure 7c) are shown in Figure 8. Data provide a broader picture of the asymmetry evolution in transmission over a broad range of forward pump powers up to 2.5 W and lengths with the optimal backward pumping (0 dB net gain). The lowest asymmetry point is found to be at 62 km, with signal power asymmetry just below 3% (97% symmetry). Further optimization is possible based on simultaneous ASE noise minimization and nonlinearity compensation [29].

**Figure 9:**
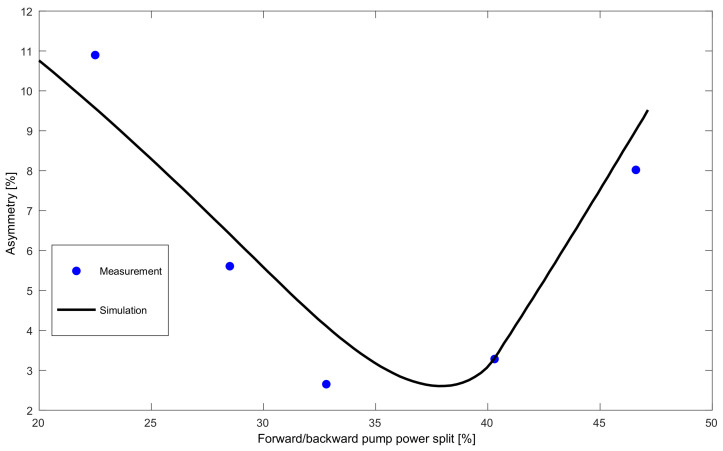
Asymmetry dependence on the forward pump power split measured at the central wavelength at 1545 nm in a 60 km span.

In order to confirm the simulation results, in Figure 9 we compare the simulated prediction and experimentally measured asymmetry vs. forward pump power split for a signal at 1545 nm in a 60 km span (the particular length was chosen due to availability of a SSMF fiber reels). The discrepancies between measurement and simulation are attributable mainly to the noisy experimental power profiles, as well as the mismatch of Raman gain and attenuation coefficients, since for consistency with previous simulations we used standard values for SSMF instead of measured coefficients.

**Figure 10:**
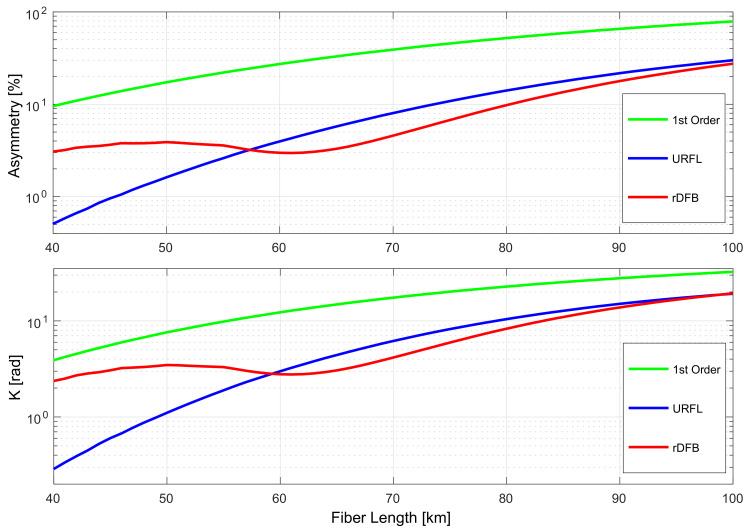
Lowest signal power asymmetry for a given length with pump powers adjusted to give zero net gain (**top**) and the accumulated residual phase shift (**bottom**) for a given amplification scheme.

Finally we numerically compare three amplification schemes shown in Figure 7 using the same method as described in Figure 8: In each case we simulate all possible FPPs adjusting backward pump to give 0 dB net gain and choose the point with the best asymmetry level (with the favor of a lower forward pump power in case if the same asymmetry is achieved for two different FPP). The results are summarized in Figure 10 below. The random DFB Raman laser amplification setup (red) achieved the lowest asymmetry levels for span lengths above 58 km. The URFL amplification option displayed better symmetry for lengths between 40 and 58 km, with optimal forward/backward pumping power ratios close to 1 in spans of up to 50 km but requiring higher contribution from the backward pump as span length grows. The random DFB configuration requires a higher contribution of the backward pump for lengths of up to 30 km, but forward/backward pump power ratio achieves close to 1 for longer spans. Optimal symmetry in first-order Raman amplification is found for backward pumping only.

The bottom of Figure 10 shows the potential accumulated residual phase shift after optimal OPC, defined as the product of the optimal asymmetry at a given distance and the corresponding nonlinear phase shift.

The combined results for high symmetry and residual phase shift results, together with resiliency to forward- pumping RIN in coherent transmission applications [29], suggest that a bidirectionally pumped random DFB laser with a single grating seems to be the best option, performance-wise, for amplification in long spans with OPC. Thus, we chose this as the option for further study.

**Figure 11:**
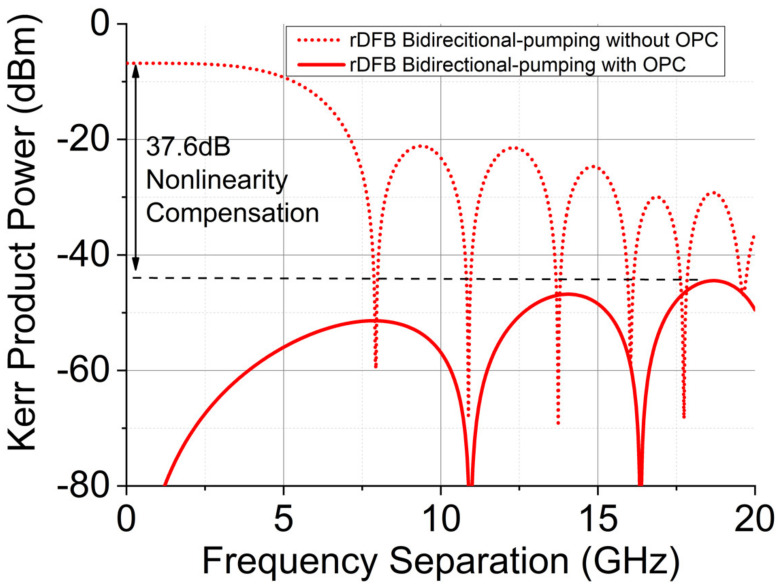
Theoretical predication of nonlinear Kerr product power as a function of frequency separation between the two CW lasers using rDFB bidirectional-pumping Raman amplification over 62 km SSMF span.

Figure 11 shows the Kerr product reduction versus frequency separation using almost symmetrical signal power profile (bidirectional pumping with random distributed feedback fiber laser). In Figure 11, without OPC, the nonlinear product power was up to −6.8 dBm near the low frequency range, but with OPC, the nonlinear product power was decreased to −44.4 dBm at 18 GHz. There was 37.6 dB nonlinear product degradation with the 97% symmetrical signal power profiles over 62 km SSMF, which was comparable with the optimized dual-order backward pumping Raman amplification over 50 km SSMF.

## 3. Optimized Distributed Raman Amplification Design over Multiple Fiber Spans

We have studied the optimization of Raman amplification schemes with backward pumping only over multiple fiber spans in [2], and this has been the only study so far regarding multiple fiber span links.

In order to investigate the impact of multi-span DRA schemes on the nonlinearity compensation, we demonstrated the signal profiles of three dual-order DRA schemes over 2 × 50.4 km SSMF spans and illustrated four-wave mixing (FWM) conversion efficiencies and inline/long-haul transmission performances.

**Figure 12:**
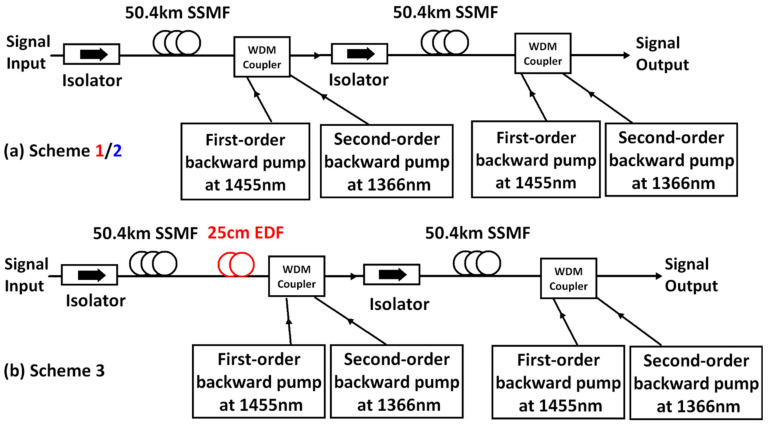
(**a**) Schemes 1 and 2: Dual-order BW-propagated pumping DRA over 2 × 50.4 km SSMF (including two pump power settings). (**b**) Scheme 3: EDF-assisted dual-order BW-propagated pumping DRA over 2 × 50.4 km SSMF.

Scheme 1 was the dual-order DRA with backward pumping which was the same as Figure 2 in each individual span that included both the first-order pump seed at 1455 nm and the second-order pump at 1366 nm [49,50]. The gain of the dual-order DRA compensated only the loss from the SSMF, and the losses of the passive components (e.g., signal pump combiners, isolators) was not compensated. In scheme 2, the experimental setup was the same as scheme 1, and the pump power for the second span also remained the same, but the 1.5 dB loss due to fiber attenuation and passive components between the two spans was compensated by more Raman power from the first span. This means that the signal output power from the first SSMF was overpowered by ~1.5 dB. In scheme 3, to compensate the loss from passive components without sacrificing the symmetry of the two-span link, a 25 cm EDF (Fibrecore M-12(980/125)) was used between the SSMF and the WDM coupler at the end of the first span as illustrated in Figure 12b, which enabled 1.5 dB amplification within this 25 cm erbium-doped fiber.

**Figure 13:**
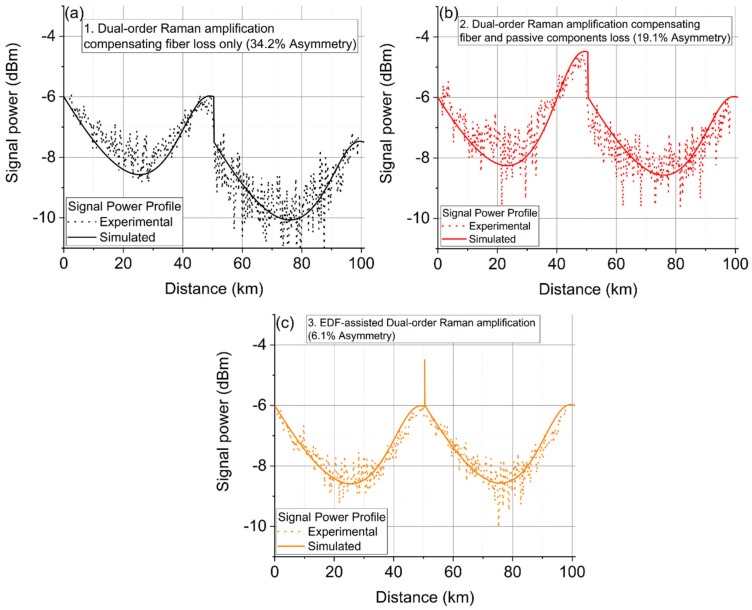
Experimentally measured and simulated signal power profiles along the fiber with three Raman amplification schemes; (**a**) DRA compensating only the fiber loss. (**b**) DRA compensating the fiber and the passive components loss between the two spans. (**c**) EDF-assisted DRA compensating fiber and the passive components loss between the two spans.

Figure 13 shows the experimentally measured and numerically simulated signal power profiles along the fiber for all three DRA schemes using the modified OTDR setup [44]. In scheme 1, the pump power was 330 mW at 1366 nm and 100 mW at 1455 nm. In the two-span link, ~1.5 dB signal power difference between two spans existed which reduced the overall signal power profile symmetry across the two spans to 65.8%, a drop dominated by the launch power difference.

In scheme 2, the pump power in the first span was 330 mW at 1366 nm and 115 mW at 1455 nm, higher than in scheme 1 to compensate the 1.5 dB loss between the spans. Matching launch powers thus improved the symmetry to 80.9%.

In scheme 3, the pump power before passing through the EDF in the first span was 330 mW at 1366 nm and 108 mW at 1455 nm. The 1.5 dB EDF amplification was powered by both Raman pumps (primarily by 1455 nm; however, the power from 1366 nm pump would be transferred to 1455 nm and contribute to the gain at low C band simultaneously) and therefore compensated the loss of passive components between spans [35]. This is different from the conventional hybrid Raman/EDFA: Instead of using 980/1480 nm laser pumps to invert the EDF fiber [51] we relied on the Raman pumps at 1366 and 1455 nm. By matching both launch power and signal evolution in the two-span Scheme enables the signal power symmetry level of 93.4% over 2 × 50.4 km spans. The symmetry level of 93.4% over two spans is also comparable to the best symmetry level of 97% over a single 50 km span using an optimized dual-order Raman amplification shown in [2].

**Figure 14:**
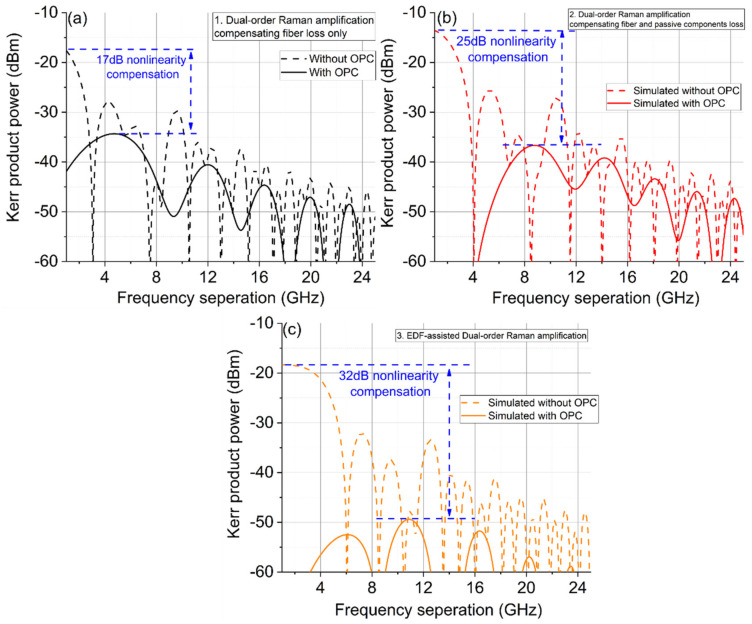
Theoretically predicated nonlinear product frequency separation without/with a mid-link OPC using different DRA schemes over 2 × 50 km spans. Dotted lines—no OPC, solid-with OPC. (**a**) No account of loss, (**b**) accounting for loss with excess Raman gain, (**c**) accounting for loss with EDF.

The theoretically predicted nonlinear product power is shown in Figure 14 as a function of frequency separation of the two lasers using the three DRA schemes. With no OPC, the nonlinear Kerr product power for all the three DRA schemes was up to −16 dBm in the low frequency region. However, because of the poor link symmetry caused by the signal power degradation between spans, scheme 1 showed the least Kerr power reduction of only ~17 dB with a mid-link OPC. Higher Kerr product power compensation (~25 dB) was achieved using the first over-pumped span. The EDF-assisted scheme generated ~1.5 dB gain to compensate the loss between spans and therefore achieved excellent signal power symmetry simultaneously for both single-span and two-span links, which showed the highest compensation (up to 32 dB) in nonlinear Kerr product power.

A numerical simulation was performed based on 2 × 2 spans (50.4 km per span, which makes ~200 km in total) using a 200 Gb/s DP-16QAM (32 GBaud, 256 Gbit/s line rate, 2^16^ PRBS length, 0.1 roll-off factor) signal centered at 194.8 THz with the three different DRA schemes (DP-16QAM signal). The nonlinear Schrödinger equations (Manakov equations) were solved using the well-known split-step Fourier method [52,53,54] with a step size of 0.1 km, in which the signal power profiles shown in Figure 13 were used. The noise from each amplifier was modeled as Gaussian noise and added to the signal after each step (0.1 km) [2] to ensure that parametric noise amplification was correctly captured. The ASE noise of EDFAs at the transmitter, the OPC and the receiver were considered in the simulations (−140 dBm/Hz noise power density). More details about the simulation parameters can be found in [2].

**Figure 15:**
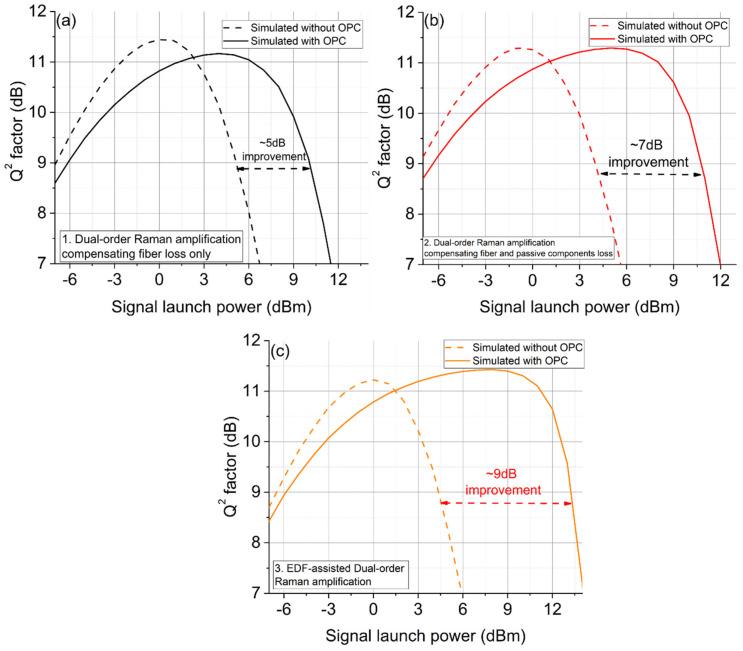
Q^2^ factors versus signal launch power in inline transmission systems without/with OPC using the optimized dual-order DRA scheme at 200 km. Dotted lines—no OPC, solid—with OPC. (**a**) No account of loss, (**b**) accounting for loss with excess Raman gain, (**c**) accounting for loss with EDF.

In Figure 15, the EDF-assisted scheme 3 shows a maximum launch power improvement of 9 dB at a fixed Q^2^ factor in the nonlinear regime, exceeding the improvement observed for the conventional DRA schemes 1 and 2 (by 4 and 2 dB), respectively. This is due to the nearly perfect signal power symmetry (>93% symmetry) from the EDF-generated gain compensating the loss between spans. However, as the overall transmission distance was ~200 km, the noise from the Raman-amplified link was limited, and therefore the results were dominated by optical noise from EDFAs in the transmitter, OPC and receiver, which obscured the Q^2^ factor benefit introduced by the nonlinearity compensation from the mid-link OPC and contributed to the small reduction in optimum Q^2^ factor with mid-link OPC [2].

**Figure 16:**
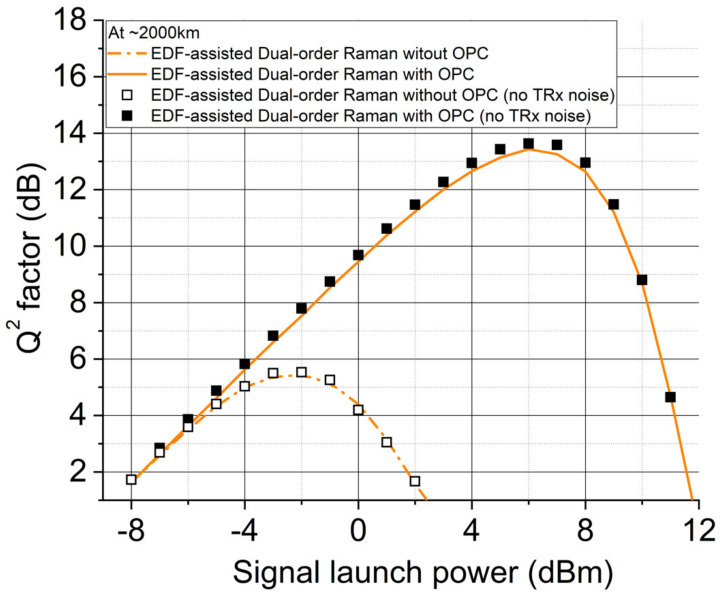
Q^2^ factors versus signal launch power in inline transmission systems without/with OPC using the optimized dual-order DRA scheme.

Figure 16 shows simulated transmission performances at 2000 km using the EDF-assisted Raman Scheme. As expected, the most symmetrical Raman scheme (Figure 13c and Figure 15c) gives ~8dB Q^2^ factor improvement (Q^2^ factor is defined as 20log10(sqrt(2).*erfcinv(2.*(BER)). In addition, the EDFA noise at the transmitter and receivers was deliberately removed for the EDF-assisted Raman scheme to evaluate the impact of such noise. In this case over 2000 km, the impact of the noise from EDFAs at the transmitter and receiver becomes negligible as the Q^2^ factor differences are very small. Thus, the accumulated noise from the Raman amplified link is more dominant compared with transceiver noise (fundamentally limited by higher-order parametric noise), and therefore the benefit of the Q^2^ factor improvement can be revealed for long-haul transmission systems with an optimized mid-link OPC [21,55]. Therefore, using the symmetrical EDF-assisted Raman link in long-haul transmission systems can improve the fiber nonlinearity compensation efficiency and the transmission performance in a mid-link OPC system.

## 4. Discussion

From our analysis in Section 2 and Section 3, to achieve the best symmetry and higher efficiency of combating fiber nonlinearity, different Raman schemes have to be considered for different span lengths. A table (Table 1) summarizing the span symmetry with Raman pumping schemes at different span lengths is demonstrated below. It is shown that for the short span length of 25 km, the first-order bidirectional Raman pumping was sufficient to achieve 97% signal power symmetry, but the length of 25 km was very short for OPC-based application (e.g., long-haul transmission systems). In addition, for this short length, the symmetry changes will be relatively small when using different Raman schemes, and bidirectional Raman pumping will introduce significant RIN-replated penalty. For the span length of around 50 km, optimized dual-order Raman pumping is required to achieve 97% span symmetry. However, for longer span length, bidirectional second-order Raman pumping would be needed as the signal gain can be generated from the start of the span. Thus, the scheme based on a random fiber laser with bidirectional second-order pumping without introducing RIN penalty was demonstrated to achieve 97% span symmetry at 62 km. Further extending the span length to 100 km, higher order bidirectional pumping would be required, but given the RIN penalty introduced from conventional Raman pumps, we had to stick to RIN-penalty-free bidirectional pumping based on a random fiber laser. In this case, the optimum span symmetry dropped to 72% at 100 km. Alternatively, we could break the 100 km into two 50 km spans, and then the problem became how to leverage the loss between the two spans. We used the EDF with Raman pumps to account for the loss between spans and improved the span symmetry from 72% (single span) to 93% (two spans).

## 5. Conclusions

We review the application of distributed Raman amplifiers with different designs for nonlinearity compensation in mid-link OPC systems. We demonstrate for single-span system with mid-link OPC that a dual-order backward-pumped Raman scheme can efficiently compensate the nonlinearity given that the pump powers are optimized to maximize the signal power profile symmetry. We show that using optimized pump powers can achieve up to 97% symmetry and 39 dB nonlinear product power reduction using a mid-link OPC. For longer span length, bidirectional Raman pumping is required to maintain a similar level of symmetry. We demonstrate that a random fiber laser amplifier is the most suitable solution for mid-link OPC WDM systems using span lengths between 60 and 100 km with the best performance at the distance of 62 km, demonstrating the Kerr product reduction up to 37.6 dB.

For multiple span systems, the optimized configurations (utilizing a 25 cm EDF) improve signal power profile symmetry and consequently enhance fiber nonlinearity compensation efficiency. This technique can compensate the loss of passive components between the spans and therefore maximize the overall signal power symmetry up to 93% in realistic multi-fiber-span link in a cost-effective manner. We demonstrate that, in the multi-span link with a mid-link OPC, using this scheme shows ~32 dB nonlinear product compensation that is at least 7 dB higher than conventional dual-order Raman schemes. We also show that, for nearly symmetrical signal power profiles, the Raman schemes in both the single-span and two-span systems give a 9 dB enhancement of the nonlinear threshold in the 200 Gb/s DP-16QAM transmission system using a mid-link OPC.

## Figures and Tables

**Table 1:** Summary of the best span symmetry with corresponding Raman schemes at different span lengths.

Span Lengths	Raman Pumping Schemes	Optimum Signal Power Profile Symmetry
25 km	First-order bidirectional pumping	97% [12]
50 km	Dual-order backward pumping	97% [23]
62 km	Second-order bidirectional pumping with random fiber laser	97% [29]
100 km	Second-order bidirectional pumping with random fiber laser	72% [29]
2 × 50 km	Dual-order backward pumping with EDF in the first span, no EDF in the second span	93% [2]

## Data Availability

Original data are available at Aston Research Explorer (https://doi.org/10.17036/researchdata.aston.ac.uk.00000534 (accessed on 16 December 2021)).
